# Pain Management in Breast Cancer Patients: A Multidisciplinary Approach

**DOI:** 10.7759/cureus.15994

**Published:** 2021-06-28

**Authors:** Lenah Sulaiman S Alhazmi, Manar Abubaker A Bawadood, Alhasan Mohammad S Aljohani, Abdulmajeed Abdullah R Alzahrani, Leena Moshref, Nora Trabulsi, Rana Moshref

**Affiliations:** 1 General Surgery, King Abdulaziz University, Jeddah, SAU

**Keywords:** pain, breast cancer, quality of life, pharmacotherapy, non-pharmacotherapy

## Abstract

Pain is a significant problem and is one of the most invalidating symptoms in breast cancer (BC) patients that would negatively affect the functional status and the Quality of Life (QoL). Pain management in BC patients requires thorough patient evaluation and critical assessment of pain. The actual cause for the pain must be recognized, so management can be tailored to each patient. This review aims to discuss various treatment modalities employed for effectively managing pain in BC patients.

Pharmacotherapy makes up the cornerstone of the management of pain in BC patients. Both opioid and non-opioid analgesics are utilized. The WHO recommends a method called “by the ladder” for managing pain in BC patients where analgesics are used in ascending order. In comprehensive pain management (CPM), non-pharmacologic therapies are gaining wide acceptance and popularity, including complementary and alternative medicine (CAM), procedural and psychosocial interventions. Procedural interventions are usually used in case of severe pain refractory to pharmacological therapy. Techniques, such as radiotherapy, neurectomy, and nerve blocks, are effective in managing cancer pain. However, CAM therapies in BC pain management need to be guided by enough scientific evidence, decision-making, and medical judgment of regulatory bodies.

BC pain management is based on careful routine pain assessments and appropriate patient evaluation both physically and psychologically. Pain control is one of the methods to improve the QoL of BC patients. Both pharmacological and non-pharmacological therapies are accessible to patients today, but they should be used with caution to minimize toxicity and increase effectiveness. The use of any pain management intervention should be based on proper scientific evidence and collective medical judgment.

## Introduction and background

Pain is a significant problem and is one of the most invalidating symptoms in breast cancer (BC) patients that would negatively affect the functional status and the Quality of Life (QoL). A painless lump is the first symptom in BC patients [1]. In advanced stages, BC patients experience severe excruciating pain, due to the involvement of inner structures, such as the muscles and ribs resulting from chest movements. The etiology of pain in BC is multifactorial, and this may be due to a) the release of inflammatory mediators, b) progression (metastasis) to other tissues such as bones, muscles, and neural structures, and c) pain related to treatment [2]. Pain can be chronic or acute or breakthrough, which may occur even after being treated. Also, pain can be generated after medical management. Chemotherapy leads to degeneration of sensory nerves, leading to neuropathic pain, [3] and radiation causes microvascular changes and nerve compression [4]. Surgery can also be the cause of pain if there was damage to the intercostobrachial nerves and the formation of neuroma [5]. The journey to cure often leads to increased psychological distress due to cancer pain, and studies show that there is a direct link between the risk of developing emotional distress with the duration and severity of the pain [6].

Pain management in BC patients requires thorough patient evaluation and critical assessment of pain. The actual cause for the pain must be recognized, so management can be tailored to each patient. Careful assessment of pain characteristics for impeccable management of BC pain, and the pain intensity reported by the patient is considered as the gold standard for routine pain assessment [7]. A comprehensive pain assessment is essential for effective pain management, and some of the most used pain assessment scales include a) the numeric rating scale, b) the visual analog scale, and c) the categorical scale [7]. Thus, effective pain management depends upon regular screening for early recognition of the pain, accurate characterization of the pain, such as onset, duration (acute or chronic), intensity, location, severity underlying pathophysiology, secondary to treatment done, breakthrough pain, etc. Also, familiarity with pain assessment techniques and determination of the type of treatment required (pharmacological or non-pharmacological). Other factors include history, previous pain management, patient prognosis, comorbidities, risk of abuse of pain medications, patient preferences, and other predictive factors, such as psychological distress.

This review aims to discuss various treatment modalities employed for effectively managing pain in BC patients.

Methodology

We conducted an integrative literature search on various Internet-based search engines (Google, Google Scholar), bibliographic databases (Pubmed, Pubmed Central, MEDLINE, Medknow, EMBASE, SCOPUS, CINHAL, AMED) for articles published from the year 1975 to 2021 using the keywords and phrases: “Management of breakthrough cancer pain” AND “Breast Cancer pain” OR “pharmacotherapy for cancer pain” OR “Medicine cancer pain” AND “Recent advances in cancer pain.” The search was limited to articles published in English. The contents with duplicity were removed, and the remaining articles were screened and assessed by the titles and abstracts. The initial search identified 174 articles. A total of 60 articles were finally considered for the review after thoroughly examining the quality and content of each one. A bibliographic management software “EndNote” (Thomson Reuters, New York, NY, USA) was used for importing the search results.

## Review

Pharmacotherapy for BC pain

Pharmacotherapy makes up the cornerstone of the management of pain in BC patients. Both opioid and non-opioid analgesics are utilized. The WHO recommends a method called “by the ladder” for managing pain in BC patients where analgesics are used in ascending order [8]. In this method, at Stage 1, non-opioids like paracetamol, ibuprofen, or acetylsalicylic acid (aspirin) are usually initiated to treat mild to moderate pain. If adequate analgesia is not attained, then Stage 2 is followed, where weak opioids like codeine and tramadol are used in case of moderate to severe pain. At Stage 3 (severe to very severe pain), stronger opioids like morphine or oxycodone are integrated if pain control is still not achieved with using weaker opioids. Adjuvant medications such as tricyclic antidepressants (TCA) (amitriptyline, desipramine), anticonvulsants (gabapentin), local anesthetics (mexilitine), corticosteroids (prednisolone, dexamethasone), and bisphosphonates (clodronate, pamidronate, zoledronic acid, and ibandronate) can also be added for managing different type of pain in any stage [8,9]. Most of the adjuvant medications mentioned here have the possibility of side effects, and it is dependent on the duration of therapy.

Most of the NSAIDs used for cancer pain management are tolerable compared to opioids; however, side effects like nausea, vomiting, gastritis, and kidney or liver dysfunction occur as shown in prospective studies [10]. These drugs should be used with caution as they exhibit ceiling effects that may cause renal and hepatic impairments and gastric problems [10].

Morphine is the most used opioid in managing severe pain in BC patients before and after surgery. It started as an oral formulation with immediate-release (IR) as the analgesic effect is obtained in 1.5 to 2 hours, then can be subsequently shifted to sustained-release (SR) as it takes 3 to 4 hours to achieve analgesia [11]. Like most opioid analgesics, there is no ceiling effect for analgesia for morphine, and hence there is no maximum safe dose [12]. There is no optimal dose for any of the opioids, but the dose that provides enough analgesia without intolerable side effects is recommended [11]. Though morphine is used in doses that range between 5 mg and 1,000 mg, using doses higher than 200 mg for management of pain should prompt the physicians to have a second thought on the diagnosis in support of morphine-resistant pain. In cases of morphine resistance, neuropathic pain should be suspected, and other management methods can be explored such as neuro-ablation and opioid rotation for improved pain control and minimal side effects [11,12]. However, another opioid member can be used when adequate analgesia is not achieved with a particular opioid, which aims to find the best medication to achieve better pain control with less toxicity [13].

A consensus concluded that the foundation treatment is opioids for debilitating chronic pain with reported tolerability and decreased side effects of fentanyl and buprenorphine use in renal patients [9]. Fentanyl, a synthetic opioid agonist, can be given to stable BC patients as oral medication or as transdermal patches and has shown same effectiveness of oral morphine according to a randomized trial. In BC patients with renal dysfunction, opioids like morphine, codeine, and hydromorphone should be used with caution as these may cause potential neurologic toxicity [14]. Transdermal patches of fentanyl are available in different doses, and the delivery rate varies from 25-100 μg/h and should be changed every 72 hours.

Oxycodone, which is superior to morphine in oral absorption and bioavailability, is found to be effective in cancer pain [15]. Oxymorphone, another semi-synthetic opioid, is used to manage moderate to severe pain in cancer patients [16]. Both opioids can be used as immediate- and extended-release formulations [17]. According to the European Association of Palliative Care, AWMF-53 Guideline for Palliative Medicine recommended the use of hydromorphone, oxycodone, morphine as the first line with similar efficacy. National Institute for Health and Care Excellence (NICE) concluded that opioid use is classified as an alternative treatment [13]. Tramadol, another weak opioid, can be used in patients with mild to moderate pain. A newer opioid, tapentadol, is available as extended-release and IR oral formulations are used to treat moderate to severe pain. In a randomized clinical trial, it is shown that no significant superiority effect of tramadol, codeine, and hydrocodone compared to one another. However, it is recommended not to mix TCA or selective serotonin reuptake inhibitors, such as fluoxetine, escitalopram, citalopram, paroxetine, and sertraline with tramadol. [17].

Opioids with a faster onset of action are essential to control episodes of breakthrough cancer pain, and in these instances, the nasal morphine-chitosan spray gives faster relief than oral morphine [15]. Opioids have more side effects compared to NSAIDS, and the most common ones include nausea, vomiting, constipation, sedation, dizziness, physical dependence, tolerance, and respiratory depression. Other less common side effects with morphine use include hyperalgesia, immunologic and hormonal dysfunction, and delayed gastric emptying [10]. Thus, it is always recommended to do proper patient screening and education of the potential side effects to get the maximum analgesia with lesser toxic effects.

In BC patients with neuropathic pain, a corticosteroid, such as dexamethasone, is also advised due to its longer duration of action and least mineralocorticoid effect [18]. Other treatment modalities include TCA like amitriptyline are utilized for effective neuropathic pain control. As level 1 evidence showed that when neuropathic pain was targeted by amitriptyline, most of the participants had more than 50% decrease in pain intensity [3]. While secondary amines like nortriptyline and desipramine are used in cases of mild to moderate pain, which also has lesser side effects as a randomized trial showed that amitriptyline was most likely to be discontinued by nearly 40% because of side effects compared to nortriptyline [19]. Clinically, neuroleptics are used to control cognitive impairment, anxiety, and psychological distress in cancer patients, but evidence shows that they can also be used to manage pain that has been unresponsive to more conventional methods [19]. The drugs commonly used to treat neuropathic cancer pain are haloperidol, olanzapine, chlorpromazine, carbamazepine, and fluoxetine [7]. Randomized double-blinded trials of less than 20 patients used casaicin for jabbing pain with good results of pain relief and a 70% success rate [3]. Noncompetitive N-methyl D-aspartate receptor antagonists like ketamine and amantadine are used as an adjuvant to opioids in cancer pain management [7,18]. Hardy et al. had demonstrated that there was no significant difference in pain management between ketamine and placebo [20]. Some of the novel pharmacological therapies that have been tried in cancer pain management include cannabinoid receptor 2 (CB2) agonist (for neuropathic pain); tetrodotoxin; botulism toxin (post-mastectomy pain] and soy isoflavones [21-23]. The multicentric study showed that participants taking cannabinoid had a decrease of pain by one-third from baseline compared to sham treatment [22].

Non-pharmacologic therapies

In comprehensive pain management (CPM), non-pharmacologic therapies are gaining wide acceptance and popularity, including complementary and alternative medicine (CAM), procedural and psychosocial interventions.

Procedural interventions are usually used in case of severe pain refractory to pharmacological therapy. Studies have shown that people who undergo axillary lymph node dissection have triple the risk of developing rest pain, unlike sentinel lymph node biopsies [5], so sentinel lymph node biopsies are recommended. Techniques such as radiotherapy, neurectomy, and nerve blocks are effective in managing cancer pain. The analgesic effect of radiation is not clear yet, but it is believed to minimize the tumor burden through decreasing osteoclasts’ activity, stimulating ossification, and killing cancer cells [24]. However, radiation therapy methods utilized for pain management is not limited to palliative radiation therapy, conventional external beam radiation therapy, novel techniques such as intensity-modulated radiation therapy, volumetrically modulated arc therapy, stereotactic radiosurgery, or stereotactic body radiation therapy, brachytherapy, or radionuclide treatment [24]. In contrast, a European study showed that nearly half of the women who undergo radiation will develop pain and lymphedema in the upcoming years [5].

The surgical management for BC pain, often referred to as ablative procedures, includes neurectomy, rhizotomy, and sympathectomy. Recent technological advancements in surgery have gain popularity in the treatment of cancer pain, such as microsurgery, stereotactic techniques and neuroaugumentative techniques for neuromodulatory processes [25]. Cordotomy is found to be effective in managing cancer pain with class 3 evidence [26]. Deep brain stimulation can also be done in BC patient who experiences a central cause of pain [26]. Among nerve blockage methods, according to meta-analysis, the pectoral nerve and the serratus plane block have been found to be effective in managing post-operative pain after BC surgery with a decreased number of analgesic request [27].

Peripheral therapies, commonly known as skin stimulation techniques, are gaining popularity and acceptance for pain control techniques, such as hydrotherapy, transcutaneous electrical nerve stimulation (TENS), massage therapy, hot-cold treatments, yoga, exercise, positioning, acupuncture, and movement restriction-resting have been used in pain control [28]. Gene therapy of NP2 enkephalin has been found to treat intractable cancer pain in the early stages of clinical trials [29].

Integrative medicine interventions, when carefully incorporated into cancer care, can be effective in pain management as these types of therapies are claimed to be free from side effects. However, CAM therapies in BC pain management need to be guided by enough scientific evidence, decision-making, and medical judgment of regulatory bodies [29].

## Conclusions

BC pain management is based on careful routine pain assessments and appropriate patient evaluation both physically and psychologically. Pain control is one of the methods to improve the QoL of BC patients. Both pharmacological and non-pharmacological therapies are accessible to patients today, but they should be used with caution to minimize toxicity and increase effectiveness. The use of any pain management intervention should be based on proper scientific evidence and collective medical judgment.
